# MCD Diet Rat Model Induces Alterations in Zinc and Iron during NAFLD Progression from Steatosis to Steatohepatitis

**DOI:** 10.3390/ijms23126817

**Published:** 2022-06-19

**Authors:** Giuseppina Palladini, Laura Giuseppina Di Pasqua, Marta Cagna, Anna Cleta Croce, Stefano Perlini, Barbara Mannucci, Antonella Profumo, Andrea Ferrigno, Mariapia Vairetti

**Affiliations:** 1 Department of Internal Medicine and Therapeutics, University of Pavia, 27100 Pavia, Italy; giuseppina.palladini@unipv.it (G.P.); lauragiuseppin.dipasqua01@universitadipavia.it (L.G.D.P.); marta.cagna02@universitadipavia.it (M.C.); stefano.perlini@unipv.it (S.P.); 2 Fondazione IRCCS (Istituti di Ricovero e Cura a Carattere Scientifico) Policlinico San Matteo, 27100 Pavia, Italy; 3 Institute of Molecular Genetics, Italian National Research Council (CNR), 27100 Pavia, Italy; annacleta.croce@igm.cnr.it; 4 Emergency Department, Fondazione IRCCS (Istituti di Ricovero e Cura a Carattere Scientifico) Policlinico San Matteo, 27100 Pavia, Italy; 5 Centro Grandi Strumenti, University of Pavia, 27100 Pavia, Italy; barbara.mannucci@unipv.it; 6 Department of Chemistry, University of Pavia, 27100 Pavia, Italy; antonella.profumo@unipv.it

**Keywords:** zinc, iron, Zn, Fe, MMPs, RECK, liver, steatosis, steatohepatitis, NAFLD, NASH

## Abstract

We evaluate the effects of the methionine-choline-deficient (MCD) diet on serum and hepatic zinc (Zn) and iron (Fe) and their relationships with matrix metalloproteinases (MMPs) and their modulators (TIMPs and RECK) as well as hepatic fatty acids using male Wistar rats fed 2-, 4- and 8-week MCD diets. Serum and hepatic Zn decrease after an 8-week MCD diet. Serum Fe increases after an 8-week MCD diet and the same occurs for hepatic Fe. An increase in hepatic MMP activity, associated with a decrease in RECK and TIMPs, is found in the MCD 8-week group. Liver Fe shows a positive correlation versus MMPs and RECK, and an inverse correlation versus TIMPs. A positive correlation is found comparing liver Zn with stearic, vaccenic and arachidonic acids, and an inverse correlation is found with linolenic and docosatetraenoic acids. An opposite trend is found between liver Fe versus these fatty acids. During NAFLD progression from steatosis to steatohepatitis, MCD rats exhibit an increase in Zn and a decrease in Fe levels both in serum and tissue associated with alterations in hepatic MMPs and their inhibitors, and fatty acids. The correlations detected between Zn and Fe versus extracellular matrix modulators and fatty acids support their potential role as therapeutic targets.

## 1. Introduction

Nonalcoholic fatty liver disease (NAFLD) is a leading cause of liver disorders, especially in individuals with features of metabolic syndrome. The mean prevalence of NAFLD worldwide is 24.1%, ranging from 13.5% in Africa to 31.8% in the Middle East, with differences among studies also related to diagnostic methods, age, gender and ethnicity [1]. 

Although its natural history has been studied over the past 20 years, NAFLD is a progressive disease whose onset and development depend on complex yet unclear factors. Several clinical and experimental studies indicate that impairment of trace element status can occur in various pathological conditions such as NAFLD. In particular, changes in zinc (Zn) and iron (Fe) have been reported in NAFLD patients [2]. Zn exhibited a negative association with the severity of the disease since higher plasma Zn levels were associated with a lower severity of NAFLD [3]. Moreover, hepatic Fe has been evaluated, showing that its overload/deposition may play a role in the NAFLD progression from steatosis to steatohepatitis [4]. A significant association between the severity of fibrosis and the increase in hepatic Fe was found in 41% of the patients evaluated [5].

Fibrosis is a disease state that results from dysfunctional wound healing in response to tissue injury, in which the extracellular matrix (ECM) production is excessive and uncontrolled [6]. A family of molecules involved in the maintenance of the ECM and processes of tissue repair is that of matrix metalloproteinases (MMPs), which were found to possess both inhibitory and stimulatory roles in fibrosis [6]. The catalytic activity of MMPs can be silenced by tissue inhibitors of metalloproteinases (TIMPs) and by MMP regulator tissue reversion-inducing cysteine-rich protein with Kazal motifs (RECK), which was the most recently identified [7]. 

The experimental models used for the identification of the NAFLD pathogenesis and its treatment by pharmacological molecules can be classified into genetic and nutritional models. Unfortunately, each model presents strengths and weaknesses with regard to their comparability to human disease conditions [8]. The methionine and choline-deficient diet (MCD) is one of the most suitable dietary patterns for NAFLD according to recent reports [9,10].

The aims of the present study are the evaluation of (1) the impact of the MCD diet on serum and hepatic Zn and Fe concentrations during the progression from steatosis to steatohepatitis and (2) the correlation of these trace elements with hepatic extracellular matrix (ECM) modulation and fatty acid composition in rats treated with the MCD diet.

## 2. Results

### 2.1. Effect of MCD Diet on Zn and Fe Levels during the Progression from Steatosis to Steatohepatitis

**Figure 1:**
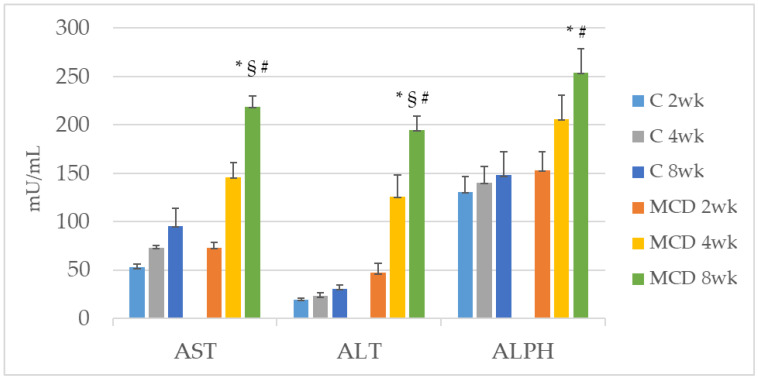
Time-course of changes in serum enzymes (AST, ALT and ALPH) using rats fed with MCD diet for 2-, 4- and 8-weeks. * *p* ≤ 0.05 versus MCD 2 wk; ^§^ *p* ≤ 0.05 versus MCD 4 wk; ^#^ *p* ≤ 0.05 versus respective control. AST, ALT and ALPH: mU/mL; weeks (wk).

We used rats treated with the MCD diet as a model of NAFLD, which progresses spontaneously to NASH. A time-dependent increase in serum levels of AST, ALT and ALPH was observed as follows: the most pronounced increase was observed in the MCD 8 wk group versus MCD 2 wk. A significant increase was also found for AST and ALT between the MCD 8 wk and MCD 4 wk groups (Figure 1). Although not statistically different, the same trend occurred for ALPH (Figure 1).

The MCD diet induced a decrease in body weight after 2, 4 and 8 weeks (Table 1) compared with their respective controls, with the lowest body weight observed at 8 weeks. The liver weight was lower in the MCD diet-treated group already after 2 weeks versus its control. The liver/body weight ratios were higher in all MCD diet-treated groups versus their respective controls. The increase was most pronounced in MCD at 8 weeks versus MCD at 2 weeks (Table 1).

**Figure 2:**
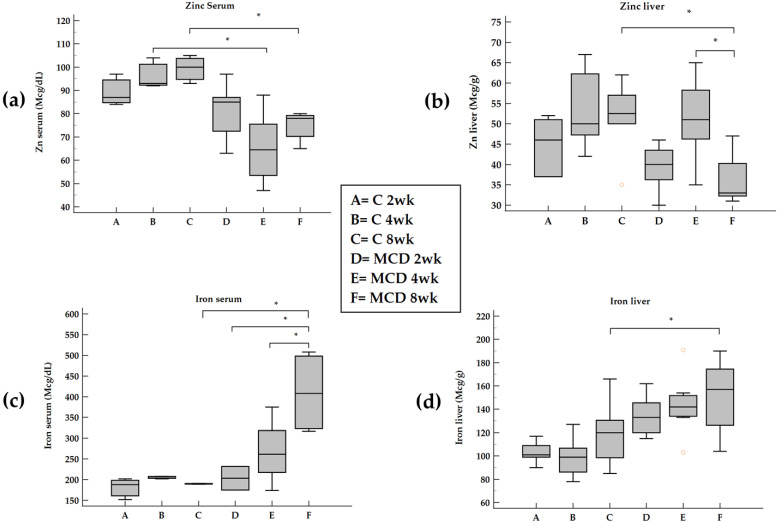
Changes in serum and tissue Zn and Fe in rats fed with MCD diet. Panel (**a**) and panel (**b**), serum and liver Zn; panel (**c**) and panel (**d**), serum and liver Fe; weeks (wk). * *p* ≤ 0.05.

During the progression from steatosis to steatohepatitis, a decrease both in serum and hepatic Zn was found after an 8-week MCD diet (Figure 2a,b). 

The levels of serum Fe increased after an 8-week MCD diet and the same trend occurred for hepatic Fe (Figure 2c,d).

### 2.2. Time-Dependent Expression of Hepatic MMPs TIMPs, RECK and Lipid Peroxidation

**Figure 3:**
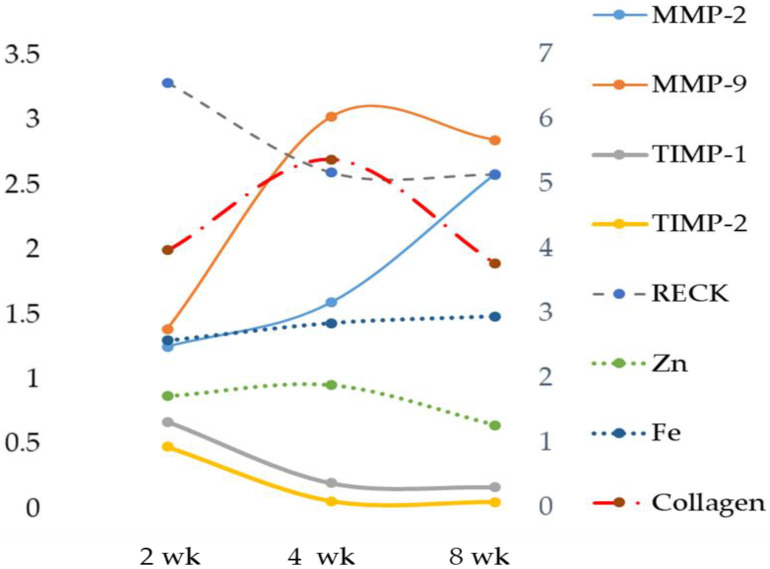
Time-dependent changes in liver Zn, Fe, MMPs, TIMPs and RECK. Zn, Fe, MMPs, TIMPs and RECK are expressed as a fold of increase/decrease in their respective control. Weeks (wk).

Tissue MMP analysis showed a significant increase in both MMP-2 and MMP-9 activity when compared with the respective controls and MCD 2 wk (Table 2). Figure 3 shows the changes in MMPs TIMPs, RECK, Zn and Fe expressed as a fold of increase/decrease in their respective controls. MMP-9 was maximally upregulated at 4 weeks. The analysis of TIMP-1 showed its decrease after 8 weeks (Table 2 and Figure 3). No difference in TIMP-2 expression emerged when comparing 2, 4 and 8 weeks (Table 2, Figure S1 and Figure 3). A time-dependent decrease was found for RECK content (Table 2, Figure S1 and Figure 3), as well as an increase in oxidative stress evaluated as TBARS and ROS formation and a decrease in GSH content. This last occurred after 2 weeks (Table 2). 

An increase in collagen, estimated in terms of the relative contribution of fluorescing fibrous proteins to the overall autofluorescence spectra recorded from liver tissue, was found (Table 2 and Figure 3).

**Figure 4:**
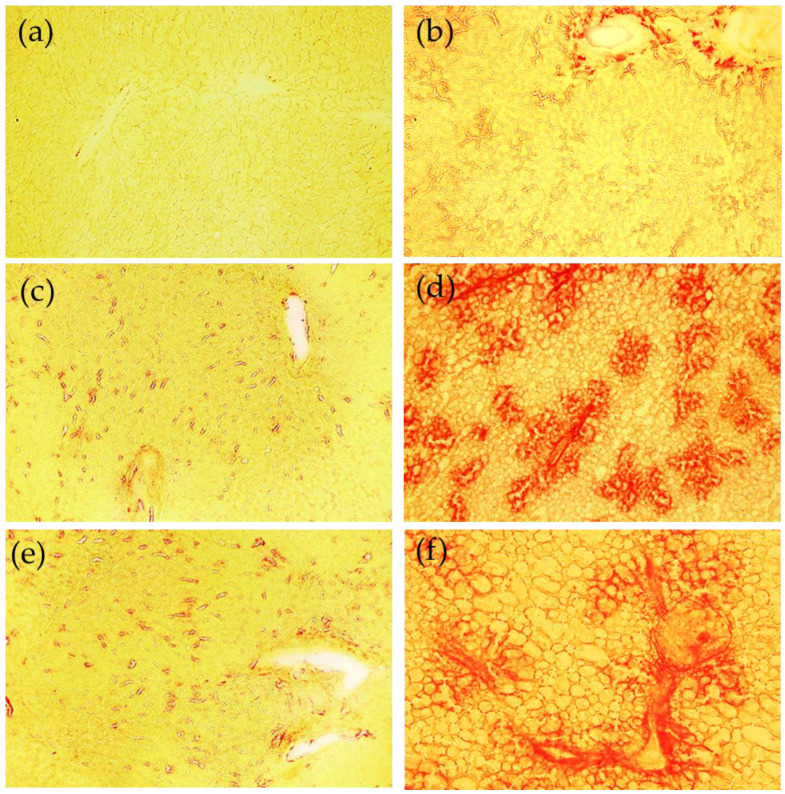
Examples of images collected from the cryostatic liver tissue sections following the Sirius red staining procedure. Fibrous collagen is evidenced as red structures. Control 2 weeks (**a**), 4 weeks (**c**), 8 weeks (**e**); MCD diet, 2 weeks (**b**), 4 weeks (**d**) and 8 weeks (**f**). Bar: 200 μm.

The histochemical Sirius red staining procedure was used to document also the time-dependent liver response to the MCD diet in terms of collagen deposition and progression to fibrosis. The changes in the patterns of liver parenchima are shown in Figure 4. The positively stained sites evidence the increase in the presence of collagen depending on the administration time of the MCD diet, from isolated foci at 2 weeks, a noticeably increased collagen deposition and growth of foci at 4 weeks, up to the formation of typical bridging fibrosis at 8 wks. At 4 weeks and 8 weeks, it can also be easily appreciated that the presence of lipid vesicles in almost all liver parenchima.

In MCD rats, correlation analysis showed that liver Zn levels were positively correlated with TIMP-1 and inversely correlated with RECK, TBARS and ROS (Table 3). No correlation was found between Zn versus MMPs and GSH.

A positive correlation was found when comparing liver Fe versus MMP-2, MMP-9, RECK, TBARS and ROS, while a remarkable inverse correlation was found with TIMP-1, TIMP-2 and GSH (Table 3). 

When comparing liver MMPs versus oxidative stress markers, a positive correlation was found when comparing MMP-2 and MMP-9 versus TBARS, while an inverse correlation was found with GSH (Table 4). MMP-2 positively correlated with ROS (Table 4).

### 2.3. Time-Dependent Changes in Serum Levels of MMPs, IL-1beta, IL-6 and TNF-alpha

**Figure 5:**
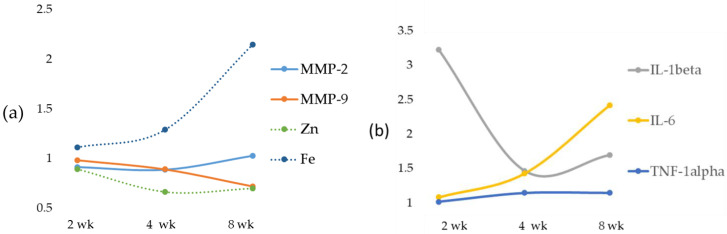
Time-dependent changes in serum Zn, Fe and MMPs (**a**) and in IL-1beta, IL-6 and TNF-alpha (**b**), expressed as a fold increase/decrease in their respective controls. Weeks (wk).

The results of serum MMP analysis showed no significant changes in both serum MMP-2 and MMP-9 (Table 5). Figure 5, panel A, shows the changes in MMPs, Zn and Fe expressed as a fold increase/decrease in their respective controls. The analysis of IL-1beta showed its increase after 2, 4 and 8 weeks when compared with the control groups; IL-1beta was maximally upregulated at 2 weeks (Table 5) and Il-6 increased at the 8-week diet treatment. No difference in TNF-alpha expression emerged when comparing 2, 4 and 8 weeks (Table 5 and Figure 5). Figure 5, panel B shows the alterations in IL-1beta, IL-6 and TNF-alpha expressed as fold increase/decrease in their respective controls.

In MCD rats, correlation analysis of serum Zn versus MMPs, IL-1beta, IL-6 and TNF-alpha showed an inverse correlation with IL-1beta and TNF-alpha (Table 6). No correlation was found for MMP-2, MMP-9 and IL-6. 

A positive correlation was only found comparing serum Fe versus IL-1beta and TNF-alpha (Table 6).

### 2.4. Time-Dependent Changes in Tissue Fatty Acids

The evaluation of liver fatty lipid composition in rats fed an MCD diet showed a significant decrease in liver palmitic acid (C16:0) at 8 wk versus 2 wk in the MCD diet group. Lower stearic acid (C18:0) was found at 2, 4 and 8 weeks versus the respective control groups. Whereas oleic acid (C18:1 n9) was significantly higher after 2 weeks, the oleic acid isomer, vaccenic acid (C18:1 n7), was significantly lower after 4 and 8 weeks. Higher levels of linoleic acid (C18:2 n6) were detected in MCD groups with the maximal concentration found after 2 weeks, and lower levels were found for arachidonic acid versus the respective controls and between MCD 8 wk versus MCD 2 wk (Table 7). An increase was found for docosatetraenoic acid and docosahexaenoic acid (DHA) in MCD for 8 weeks (Table 7). A significant decrease in docosapentaenoic acid C 22:5 n3 also occurred after a 4- and 8-week MCD diet versus MCD 2 weeks (Table 7). 

**Figure 6:**
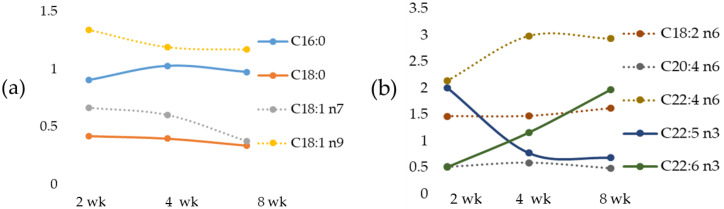
Time-dependent changes in liver fatty acids expressed as a fold increase/decrease in their respective controls. Tissue SFA and MUFA, panel (**a**); tissue PUFA panel (**b**). Weeks (wk).

Furthermore, Figure 6. shows the time-dependent alterations in fatty acids expressed as a fold of increase/decrease in their respective controls.

In MCD rats, a positive correlation was found comparing liver Zn versus stearic acid, vaccenic acid, arachidonic acid and docosapentaenoic acid (Table 8); on the contrary, an inverse correlation between linoleic acid and docosatetraenoic acid was reported (Table 8). 

An opposite correlation, compared with that observed for tissue Zn, occurred between liver Fe versus stearic acid, vaccenic acid, arachidonic acid and with docosatetraenoic acid (Table 8).

## 3. Discussion

Our results show that, in rats treated with an MCD diet, the progression from steatosis to steatohepatitis significantly affects serum and tissue levels of Zn and Fe; we also documented the upregulation of MMPs and a decrease in TIMPs and RECK, as well as the existence of significant correlations between Zn and Fe versus both molecules involved in the control of the ECM and pro-inflammation markers such as IL-1beta and TNF-alpha. In addition, we found significant correlations between Zn and Fe versus specific fatty acids.

NAFLD is the most common cause of chronic liver disease, and the activation of inflammatory pathways could contribute to disease pathogenesis. In the estimation of the World Health Organization (2016), around 1.9 billion adults (people over 18 years of age) were overweight, and more than 600 million were obese [11]. It is expected that, by 2030, more than 2.16 billion people will be overweight and 1.12 billion obese [12], of which one-fourth of the adult population will be affected by NAFLD [1]. Thus, there is an urgent need for effective interventions to prevent the NAFLD progression from steatosis to steatohepatitis [2]. 

The development of an appropriate animal model is crucial in developing a treatment for this rapidly growing disease. The mouse/rat model diets can be identified in the following two types: the MCD diet and the Western-style diet (WD). In the MCD diet, the absence of methionine leads to hepatic injury, inflammation and fibrosis, and the deficiency of choline leads to macrovesicular steatosis [13]. In the MCD-fed animals, the absence of systemic insulin resistance and the presence of hepatic insulin resistance were found [14]; thus, this model may help to delineate NASH pathogenesis in the absence of confounding parameters and to identify the involvement of non-insulin resistant mechanisms [15]. Furthermore, the MCD-fed animal model provides the histological hallmark of NASH because of its vulnerability to transition from simple steatosis to steatohepatitis [16], becoming a widely used model for the study of the progression of NASH [17].

In detail, choline, an essential nutrient, is necessary for the de novo synthesis of phosphatidylcholine, needed for the export of triglycerides out of hepatocytes via very-low-density lipoprotein packaging [18]. Humans can become depleted of choline and previous data showed that choline deficiency in patients that need parenteral nutrition results in the development of hepatic steatosis, similar to various animal models [19]. Furthermore, methionine is an essential amino acid that plays a key role in many cellular functions because it is used for protein synthesis and as an intermediate in S-adenosylmethionine (SAM) and GSH synthesis, two important metabolites in cellular homeostasis and hepatocyte function [20].

Here we report that in rats treated with an MCD diet, a time-dependent decrease in Zn and an increase in Fe occurred both in serum and in the liver. Furthermore, the co-existence of Fe and Zn changes can contribute to the liver’s development of fibrosis as supported by the relationships with these two oligo-elements versus molecules involved in ECM modulation.

### 3.1. Zn and Fe and MMPs

Serum and hepatic Zn concentrations are decreased in chronic liver diseases, and Zn depletion has been suggested as a cause of hepatic fibrosis [21]. The probable underlying mechanism is that the MCD diet induces an inflammatory state in which the expression of gelatinases increases [22]. 

Inflammation has been considered the main cause of chronic diseases, and Zn can influence the production and signaling of numerous inflammatory cytokines in a variety of cell types [23]. Studies evaluating simultaneously circulating cytokines and Zn status showed that the reduced circulating Zn correlates with increased IL-6, IL-8 and TNF-α levels [24]. In support of these findings, in our data, an inverse correlation between serum Zn and IL-1beta and TNF-alpha was found. On the contrary, no correlation was found between Zn and IL-6. During the progression from NAFLD to NASH, we observed an opposite trend of Zn in serum and tissues with a switch at 4 weeks attributable to an inverse relationship between hepatic Zn content and serum Zn levels [25], with a mechanism probably modulated by interleukins. In fact, in infectious diseases, interleukins released from activated phagocytic cells have been shown to reduce plasma Zn concentrations by redistributing it from plasma to the liver [23]. Zn deficiency caused by chronic liver disease also evokes metabolic alterations such as insulin resistance, Fe overload and hepatic steatosis [26].

Zn also competes with Fe and a weak inverse correlation between serum Zn and ferritin was found in HCV-related chronic liver disease patients associated with NAFLD [27]. On the other hand, it has been established that Zn and Fe may compete for access to transporters as follows: divalent metal transporter 1 (DMT1) is a possible candidate Zn-Fe transporter, as well as Zip14, is another possible candidate for transport of both Zn and Fe in the liver [28,29]. Zip14 has been documented to mediate Fe not bound to transferrin in the liver, which could lead to Fe overload [30]. Both in vivo and in vitro experiments in the liver demonstrated that Zn transporter Zip14 is regulated by IL-6, likely contributing to the hypozincemia observed during inflammation [31]. Zip14, one of the major cytokine-responsive transporters localized at the plasma membrane of hepatocytes, could be thus involved in the MCD diet model as an increase in IL-6 occurs. Although in tissues different from the liver, IL-1beta has also been demonstrated to downregulate a number of Zn-transporters, most strikingly ZnT8 [32].

The role of Zn as an anti-inflammatory and antioxidant agent would justify the reduction of the expression of MMP-2 and MMP-9 [22]. Vice versa, during NAFLD progression from steatosis to steatohepatitis, a Zn decrease is correlated with an increase in gelatinase activity [33]. The reported data in our MCD model showed a significant increasing trend in both liver MMP-2 and MMP-9 when compared with their respective control and MCD 2 wk, while the result of serum MMP analysis showed no significant change in gelatinase activity. In spite of the MMP-2 and MMP-9 increase, no significant correlation was found between liver or serum Zn and gelatinases. Concurrently, a significant reduction of TIMP-1 and a tendency toward reduction of TIMP-2 were found. In addition, the novel matrix metalloproteinase regulator, RECK [34], was significantly reduced after 8 weeks of the MCD diet. A great deal of interest has been shown in tissue inhibitor metalloproteinase (TIMP) levels as non-invasive biomarkers for the diagnosis of NAFLD [35]. 

In the observational one-point study by Abdelaziz, the authors evaluated the status of TIMP-1 and TIMP-2 in patients with NAFLD and compared these levels with those of obese and control patients [35]. They showed that serum levels of both TIMPs were significantly elevated in NASH patients as compared to the control group. These results are not consistent with ours. Both TIMP-1 and TIMP-2 had significant diagnostic ability in detecting advanced liver disease but the status of TIMP-1 and TIMP-2, in the algorithm of NAFLD diagnosis, is yet to be established. Furthermore, we evaluated TIMPs in the tissue, not in the plasma, and it is known that TIMPs prevent cellular MMP’s fibrinolytic action in a stoichiometric manner [36].

Oxidative stress plays an important role in the pathophysiology of NAFLD/NASH. For the purpose of evaluating lipid peroxidation, various markers of oxidative stress and related antioxidants have been evaluated over the years [37]. Among the oxidative stress biomarkers examined, TBARS, MDA, CYP2E1 and 4-HNE were found to be incisive; their steady increase during the transition from NAFLD to NASH has been reported in both clinical and experimental settings [38]. In the present study, the increased TBARS and ROS values of the MCD rats correlated negatively with tissue concentrations of Zn as expected. In addition, TBARS and ROS were also correlated with gelatinases.

We documented, using rats submitted to the MCD diet, a time-dependent increase in serum Fe that occurs by a systemic insulin-independent mechanism. This finding is in agreement with a previous study on the MCD model where hepatic Fe overload was associated with a trend toward increased perivenular fibrosis [39].

The role of hepatic Fe in the progression of steatosis to steatohepatitis remains controversial, but the hypothesis that Fe may play a role in the pathogenesis of NASH is increasingly emerging [40]. 

The mechanism for this progression is not yet known, but a “two hit theory” is generally accepted. The first hit is considered to be insulin resistance, resulting in an excessive accumulation of fat in the liver. The second hit is the generation of oxidative stress by a number of factors, including hepatic Fe overload, which leads to cytotoxicity and inflammation [41]. However, further studies are needed to define the contribution of hepatic Fe to severe disease progression [42].

Fe overload has been reported in 30–70% of patients with non-alcoholic fatty liver disease (NAFLD) and non-alcoholic steatohepatitis (NASH) [43]. Fe could potentially play a supporting role in lipid peroxidation and fibrogenesis during the development and progression of NASH by inducing fibrosis-promoting signals in the parenchymal (hepatocytes) and non-parenchymal cells (hepatic stellate cells-HSCs, liver sinusoidal endothelial cells, Kupffer cells) [4]. Liver injury stimulates the non-parenchymal cells to secrete several profibrogenic cytokines. Pro-inflammatory mediators cause quiescent HSCs to differentiate into myofibroblast-like cells to produce extracellular matrix (ECM) components, which are subsequently degraded by matrix metalloproteinases (MMPs). In turn, MMP activity is inhibited and modulated by specific tissue inhibitors (TIMPs), produced by the activated HSCs. Subsequently, the activated HSCs either undergo apoptosis and/or revert to their original quiescent phenotype, thereby terminating a well-regulated and reversible healing process [44]. Prolonged liver injury via chronic inflammation, infection and/or oxidative stress leads to continuous stimulation of the wound healing mechanism whereby the HSCs remain persistently activated, leading to excessive deposition of ECM [45]. The fibrotic responses are collectively mediated by multiple mechanisms. 

Excess Fe accelerates the Fenton reaction to generate ROS, which can oxidize lipids, proteins and nucleic acids, thereby promoting fibrosis initiation and/or fibrosis progression. ROS, in turn, induces lipid peroxidation [46]. The by-products of lipid peroxidation detected in the liver of Fe-loaded rats can act as profibrogenic stimuli [47]. In support of this, we detected a significant and positive correlation between liver Fe and TBARS. Sensitized hepatocytes produce a pool of pro-fibrogenic and pro-inflammatory mediators such as IL-6, Il-1beta and TNF-alpha. Cytokines, particularly pro-inflammatory cytokines, have been shown to play an important role in the pathological progression of NASH [48]. This was evident in our MCD-induced NASH rat model due to the significant increase in the levels of the pro-inflammatory cytokine IL-6.

Another mechanism involved is Fe-mediated ECM remodeling. Studies have shown that Fe promotes collagen crosslinking. This form of collagen is more resistant to proteolytic degradation by MMPs [49]. However, a previous in vitro study excluded Fe as a major participant in collagen crosslinking since the Fe chelator deferoxamine did not alter collagen modifications [50]. As a result, to date, the exact effect of Fe on collagen maturation is still unclear.

Our data suggest that Fe plays a role in matrix degradation by stimulating some metalloprotease activities. In our MCD model, a positive correlation was found comparing hepatic Fe versus gelatinases MMP-9 and MMP-2. In particular, during hepatic fibrosis, the metalloproteinase mainly expressed is MMP-2, and our results are in accordance with data obtained by Gardi et al. [51]. We also evaluated the inhibition of MMPs activity by measuring both the expression of TIMPs and RECK, a novel matrix metalloproteinase regulator [34]. A significant decrease was detected for TIMP-1 and TIMP-2, and a time-dependent decrease was found for RECK. This newly discovered MMP inhibitor does not show similarities to TIMPs in amino acid sequence and can inhibit at least three members of the MMP family as follows: MMP-2, MMP-9 and MT1-MMP, playing an important role in the process of degradation of ECM. The statistical analysis showed a strong inverse correlation between Fe and specific MMP inhibitors TIMP-1 and TIMP-2 and a significant positive correlation with RECK, despite the latter’s role being comparable to that of TIMPs. Anyway, to date, direct evidence of the role of Fe on RECK is lacking.

### 3.2. Zn and Fe and Fatty Acids

Linoleic acid (LA), an omega-6 fatty acid and α-linolenic acid (ALA), an omega-3 fatty acid, are considered essential fatty acids (EFA) because they cannot be synthesized by humans, and a relationship between Zn and EFA deficiency has been proposed [52].

We documented, in the MCD diet rat model, a time-dependent change of the most important fatty acids that occurred early in fatty acid accumulation, in agreement with data previously reported in mice fed with the MCD diet [53]. We have documented a tissue increase in DHA after an 8-wk MCD diet, also found in the liver of high-fat diet mice [54]. Of note, the significant correlation between Zn and fatty acid composition reported in the present study suggests that in the presence of a consistent Zn decrease a change in lipid composition occurs; on the contrary, a moderate Zn deficiency, as that observed in weanling rats fed diets rich in Cocoa Butter or Safflower Oil, was not able to alter significantly liver lipid concentrations and fatty acid composition [55]. We also documented in the MCD rats a correlation between Fe versus the most relevant fatty acids. Linoleic acid, an EFA, was inversely correlated with Zn and positively with Fe. Particularly, an increase in LA occurred with a peak at 2-wk MCD diet. 

Arachidonic acid (AA), as a pro-inflammatory precursor, is associated with metabolic disorders [56]. We documented low AA in the livers of MCD 2 wk rats and a significant increase in the MCD 8 wk group; our results are in agreement with those observed in mice treated with the MCD diet [53]. Similar results have been also found in rats during high-fat diet (HFD)-induced NAFLD development [56]. Based on these results, we may propose that changes in AA content may be an early indicator of inflammation in NAFLD progression. 

Docosapentaenoic acid, or DPA, has been reported to have a major role in reducing inflammation [57]. In the MCD diet model, a strong reduction in DPA has been found at 8 weeks, and this event is associated with an increase in IL-6. 

A recent study, based on combined classical histology, pathological evaluation and gene expression analysis, reported that the MCD diet model recapitulates the liver manifestation of human NASH [14]. Although the MCD diet does not provoke systemic insulin resistance, which is important in the pathogenesis of NAFLD in humans, this model may be potentially used for the investigation of the lipotoxic effects of fatty acids. Moreover, the MCD diet model produces reproducible and rapid changes similar to those observed in human NASH, and changes in fatty acids already occurred after 2 weeks. 

The findings of this study reinforce the roles of the high prevalence of Zn deficiency and Fe overload among subjects undergoing NAFLD, also found in the MCD experimental model. The results obtained indicate that the assessment of Zn and Fe status should provide a standard parameter of nutritional status screening in NAFLD patients. Although further research is required, Zn supplementation and a control of Fe overload in NAFD patients might be beneficial for the prevention and attenuation of adverse health outcomes.

Diet influences the progression of NAFLD; following a western diet or simply a high-fat diet may contribute to the worsening of NAFLD and further progression to NASH and cirrhosis in later stages. A systematic review found that saturated fatty acids, trans-fats, animal proteins and simple sugars have a harmful effect on the liver [58]. Dietary changes can slow these events and prevent lasting liver damage. For example, Zn supplementation has beneficial metabolic effects in patients with NAFLD risk factors. Plasma Zn levels should be evaluated in patients with NAFLD and NAFLD risk factors, specifically in those with advanced hepatic fibrosis/cirrhosis [59]. In addition, the Mediterranean diet is the gold standard for both treatment and prevention [60]. This dietary pattern is characterized by a high intake of olive oil, which is rich in monounsaturated fat. It has also been shown that diets enriched with omega-3 polyunsaturated fatty acids (PUFA) ameliorate steatohepatitis, together with a reduction in intrahepatic triglyceride content [61]; this is strongly recommended since a lower consumption of omega-3 PUFA was found in NASH patients [62].

## 4. Materials and Methods

### 4.1. Animal Model

The animal model used was approved by the Italian Ministry of Health and the Pavia University Animal Care Commission (Document number 2/2012). Male Wistar rats were fed with the Methione and Choline Deficient diet (MCD) (Laboratorio Dottori Piccioni. Gessate, MI, Italy) [63] for 2, 4 and 8 weeks (week 2: *n* = 5, week 4: *n* = 5 and week 8: *n* = 5). As controls, rats fed for 2, 4 and 8 weeks with a choline- and methionine-containing diet, isocaloric with MCD, were used (*n* = 12). Rats were anesthetized (Pentobarbital i.p. injection, 40 mg/kg), the livers were exposed and the tissue specimens collected from the left lobe, immediately frozen in liquid nitrogen and stored at −80 °C until being processed for biochemical assays, or cut at cryostat for histochemical assay (tissue sections 8 μm thick, −24 °C; Leica CM 1850 cryostat model, Leica Biosystems Italia, Milano, Italy) [64]. 

### 4.2. Serum and Tissue Measures

Liver injury was assessed by serum levels of alanine transaminase (ALT), aspartate transaminase (AST), alkaline phosphatase (ALPH), total and direct bilirubin, assayed by commercial kit (MERK Serono, Rome, Italy). Serum levels of glucose, p-cholinesterase, cholesterol and triglycerides were also evaluated by commercial kit (MERK Serono, Rome, Italy). Serum levels of inflammation markers, TNF-alpha (Cod865000192), IL-1beta (Cod670040096) and IL-6 (Cod670010096) were determined by using ELISA Kits (Bertin Bioreagent, Alfatech S.p.A, Genova, Italy). The hepatic concentration of total glutathione (GSH, nmol/mg prot) was measured by an enzymatic method (Cayman Chemical Co., Ann Arbor, MI, USA). The extent of liver lipid peroxidation in terms of thiobarbituric acid reactive substances (TBARS, nmol/mg prot) formation was measured according to the method of Esterbauer and Cheeseman [65]. The TBARS concentrations were calculated using malondialdehyde (MDA) as standard ROS (A.U.) were quantified by the DCFH-DA method based on the ROS-dependent oxidation of DCFH to DCF, as already described [66]. Protein content was assayed by the method of Lowry et al. [67].

### 4.3. Serum and Hepatic Levels of Zn and Fe

Samples, liver (ca. 100 mg each accurately weighed) and serum (500µL), were digested with 3 mL of Trace-SELECT^®^ Ultra ultrapure HNO3 (65% *w*/*w*) plus 1 mL of H_2_O_2_ (30% *w*/*w*) under reflux for 15 min. After cooling, the contents were evaporated to a small volume (ca. 0.5 mL), diluted to 10 mL with ultrapure water in calibrated polypropylene tubes and filtered at 0.22 µm before analysis. Five-point calibration curves for Fe and Zn were generated in the range 10–5000 µg/L from certified multistandard solution by MERK Serono (Rome, Italy) and acidified with 0.5% HNO_3._ Method detection and quantification limits (MDLs, MQLs, respectively) were obtained from the instrumental detection and quantification limits (IDLs, IQLs, respectively) calculated using the residual standard deviation (Sy/x) of the linear regression parameters as (3.3 × Sy/x)/slope and (10 × Sy/x)/slope, respectively) and are referred to the overall procedure. Reagent blanks were prepared following the same procedure applied to samples. Measurements were performed by a Thermo Scientific iCAP 7000 duo series Inductively coupled plasma optical emission spectroscopy (ICP-OES) instrument, equipped with a quartz torch and a Charge Injection Device detector.

### 4.4. Hepatic Lipid Extraction and Quantification

Hepatic lipid quantification was performed according to Lyn-Cook et al., 2009 [68]. Briefly, frozen tissues (50–70 mg each) were homogenized in 200 µL of water. Lipids were extracted by adding 1 mL chloroform-methanol (2:1) and samples incubated for 1 h at room temperature with intermittent agitation. After centrifuging at 3000 rpm for 5 min at room temperature to separate the lipid-containing lower phase, that was transferred to a clean tube and N2-dried. Pellets were re-suspended in 100 µL of 100% ethanol. 

Fatty acid profile has been analyzed by a ThermoFisher Scientific DSQII GC/MS system (TraceDSQII mass spectrometer, TraceGCUltra gascromatograph), Xcalibur MS Software Version 2.1 (including NIST Mass Spectral Library (NIST 08) and Wiley Registry of Mass Spectral Data 8th Edition for assignment of chemical structures to chromatographic peaks) [64]. To proceed to fatty acid assay, 5 µL aliquots of rat liver extracts were dissolved in 1 mL methanolic HCl (2N) into reaction vials. The vials were capped and heated at 70–80 °C for 4 h. The samples were allowed to cool, then dried under nitrogen stream. Later 250 µL of dichloromethane was added and 1 µL aliquot was sampled for analysis. Dichloromethane alone was used as blank to avoid carryover from previous analysis. The reference standard Marine Oil FAME Mix from Restek S.r.l. 20063 Cernusco sul Naviglio MI, Italy (cat. 35066) was used to identify and quantify the fatty acids. The multianalyte standard solution was 10–160 µg/mL in hexane. Each identified peak was expressed as relative percentage area of total methylated fatty acids (FAME). 

### 4.5. Gelatin Zymography

Protein extraction from snap-frozen samples and gelatin zymography were performed as described previously [69]. To detect MMP lytic activity, samples were homogenized in an ice-cold extraction buffer and protein content was normalized by a final concentration of 400 μg/mL in the sample loading buffer (0.25 M Tris-HCl, 4% sucrose *w*/*v*, 10% SDS *w*/*v* and 0.1% bromphenol blue *w*/*v*, pH 6.8). After dilution, the samples were loaded onto electrophoretic gels (SDS-PAGE), containing 1 mg/mL gelatin, under non-reducing conditions. Gels were run at 15 mA/gel through the stacking phase (4%) and at 20 mA/gel for the separating phase (10%), at 4 °C in a running buffer. After the run, gels were washed twice and incubated for 18 h at 37 °C in incubation buffer. At the end of incubation gels were stained with Coomassie Blue to reveal zones of lysis. The zymograms were analyzed by a densitometer (GS 900 Densitometer BIORAD, Hercules, CA, USA).

### 4.6. Western Blot Assay

Liver tissue samples were homogenized in an ice-cold Lysis Buffer supplemented with Protease Inhibitor Cocktail and centrifuged at 15,000× *g* for 10 min. The collected supernatant was stored at −80 °C. Samples of liver extracts containing the same number of proteins were separated in SDS-PAGE on 7.5% acrylamide gels and transferred to PVDF membrane. Unspecific sites were blocked for 2 h with 5% Bovine Serum Albumin (BSA) in TBS Tween (20 mM Tris/HCl, 500 mM NaCl, pH 7.5, 0.1% Tween 20) at 4 °C. The membranes were incubated with primary antibodies overnight at 4 °C, under gentle agitation. Primary antibodies against mouse monoclonal alpha tubulin (DM1A) and mouse monoclonal anti-RECK were used at 1:1000 dilution. Rabbit polyclonal anti-TIMP-1 andTIMP-2 were used at 1:200. Membranes were washed in TBS Tween (Na2HPO4 8 mM, NaH2PO4-H2O2 mM, NaCl 140 mM, pH 7.4, 0.1% Tween 20) and incubated with peroxidase-conjugated secondary anti-Rabbit or anti-Mouse antibodies at a 1:2000 dilution. The membranes were then stripped and incubated with tubulin monoclonal antibody (1:5000) and subsequently with anti-mouse (1:10,000) to assess uniformity of gel loading. RECK was bought from Santa Cruz Biotechnology. Mouse monoclonal antibodies against TIMP-1 and TIMP-2 were purchased from Thermo Fisher Scientific (Waltham, MA, USA). Immunostaining was revealed with BIO-RAD Chemidoc XRS+ visualized using the ECL Clarity BIO-RAD (Segrate, MI, Italy). Band intensity quantification was performed by BIO-RAD Image Lab Software™6.0.1., (Segrate, MI, Italy) and autoradiograms showing statistically significant differences in terms of gel-loading homogeneity were excluded from the following biomarkers analyses.

### 4.7. Liver Fibrosis

Progression of fibrosis in the liver was assessed by estimating the autofluorescence of collagen by means of in vivo spectrofluorometric analysis of liver tissue. The autofluorescence emission was measured by means of an optic-based system and a single fiber optic probe (diameter 300 μm, Fiberlan, Milan, Italy) to guide the excitation light to the exposed liver and to collect and guide the emission signal to the detection system. The optical system ensured the optical coupling by means of a beam splitting device (Oriel Instruments, Stratford, CT, USA) mounting a 390 nm dichroic mirror (Chroma Technology Corp, Rockingham, VT, USA) to select the excitation light (366 nm, 5.0 W LED, Fraen Corporation, Trivolzio, PV, Italy) and a barrier filter (GG 395, Oriel, Newport Corporation, Irvine, CA, USA) to select the autofluorescence signal. The fiber optic measuring probe was gently inserted into the liver tissue to collect the autofluorescence signal. Each spectral scan acquisition lasted 400 ms, and 10 scans were repeated for each measuring site. Spectra were recorded in the 400–750 nm range by means of the PMA11 optical multichannel analyzer (Hamamatsu Photonics Deutschland GmbH, Herrsching am Ammersee, Germany). 

The signal ascribable to collagen was estimated from the overall autofluorescence emission of the liver by means of a curve-fitting procedure 8PeakFit: SPSS Science, Chicago, IL, USA) already described in detail [70]. In brief, the processing was based on the finding of the true absolute minimum value of the sum of squared deviations between each measured spectrum and the curve resulting from the sum of the spectral functions, described by half-Gaussian Modified Gaussian (GMG) parameters typically representing each fluorescing species of liver, combined in order to find the relative contributions of each fluorophore and thus of collagen. The contribution of collagen was given as percentage of the overall spectral area normalized to 100 a.u. 

Fibrous collagen was also evidenced by means of a histochemical procedure based on the Sirius red (Direct Red 80) staining [71]. Cryostatic tissue sections collected on glass slides were air-dried (48 h) before to immersed for 1 h in the staining solution (0.1% of Sirius red in picric acid-saturated aqueous medium). After rinsing in two changes of water (acidified, 0.5% acetic acid) slides were dehydrated and mounted for observation at microscope under bright field conditions (Olympus BX53 model, Olympus Optical Co. GmBH, Hamburg, Germany) by using the UPlanFL 20X (na 0.50) and UPlanFL 40X (na 0.75) objectives.

### 4.8. Statistical Analysis

All data were statistically analyzed using MedCalc Statistical Software (version 18.11.3) and averaged values were presented as means ± SEM or median where indicated. Statistical analysis was performed with one-way ANOVA and a Tukey’s ANOVA posttest was used for multiple comparisons. When data distribution was not normal Kruskal–Wallis and Dunn’s test was used. Association between parameters was evaluated according to Pearson correlation for normally distributed variables or Spearman rank correlation when the variables are not normally distributed. The value of *p* < 0.05 was used as the level of significance.

## 5. Conclusions

We documented, in the MCD diet model, alterations in Zn and Fe, both in serum and liver, during the NAFLD progression from steatosis to steatohepatitis. The consequences of Zn deficiency and Fe overload have been found to affect the cellular processes that result in pathological changes such as ECM remodeling, inflammation and fatty acid modifications. The patterns of changes likely reflect compensatory and pathological changes associated with liver Zn deficiency and Fe overload and provide a window into these processes. 

## Figures and Tables

**Table 1:** Effects of MCD diet on rat liver weight, body weight and liver/body weight ratio.

	C 2 wk	MCD 2 wk	C 4 wk	MCD 4 wk	C 8 wk	MCD 8 wk
Liver weight	15.12 ± 0.71	11.44 ± 0.43 ^#^	16.39 ± 0.95	14.62 ± 0.87	16.17 ± 0.94	13.49 ± 0.93
Body weight	355.45 ± 9.94	239.64 ± 6.13 ^#^	448.11 ± 16.95	269.17 ± 16.19 ^#^	489.67 ± 15.50	220.77 ± 5.92 ^#,$^
Liver/body weight (%)	4.25 ± 0.11	4.78 ± 0.14 ^#^	3.65 ± 0.12	5.51 ± 0.43 ^#^	3.29 ± 0.13	6.11 ± 0.38 ^#,^*

* *p* ≤ 0.05 versus MCD 2 wk; ^$^ *p* ≤ 0.05 versus MCD 4 wk; ^#^ *p* ≤ 0.05 versus respective control. Liver weight. and body weight: g; weeks (wk).

**Table 2:** Changes in liver MMPs, TIMPs, RECK, TBARS, ROS, GSH and collagen in rats fed with MCD diet.

	C 2 wk	MCD 2 wk	C 4 wk	MCD 4 wk	C 8 wk	MCD 8 wk
MMP-2	224.79 ± 33.38	282.50 ± 21.08	247.71 ± 28.71	396.04 ± 57.41	213.00 ± 27.95	491.01 ± 40.80 *^,#^
MMP-9	31.56 ± 13.37	43.95 ± 10.44	43.58 ± 5.99	132.34 ± 30.34 ^#^	38.91 ± 4.89	110.74 ± 8.75 *^,#^
TIMP-1	0.92 ± 0.14	0.55 ± 0.11 ^#^	1.23 ± 0.15	0.25 ± 0.06 ^#^	1.15 ± 0.18	0.24 ± 0.05 *^,#^
TIMP-2	0.62 ± 0.13	0.25 ± 0.03 ^#^	0.65 ± 0.07	0.18 ± 0.05 ^#^	0.56 ± 0.13	0.26 ± 0.05 ^#^
RECK	0.31 ± 0.09	1.02 ± 0.07 ^#^	0.25 ± 0.01	0.65 ± 0.16 ^#^	0.24 ± 0.06	0.62 ± 0.09 *^,#^
TBARS	0.06 ± 0.01	0.13 ± 0.03 ^#^	0.06 ± 0.01	0.73 ± 0.12 ^#^	0.08 ± 0.01	2.02 ± 0.35 ^#,^*^,$^
ROS	33,765.16 ± 9574.98	97,357.25 ± 6702.58	14,118.83 ± 1599.29	351,301.40 ± 57,221.08	48,229.00 ± 1778.59	624,931.40 ± 30,738.30
GSH	37.31 ± 1.49	19.33 ± 1.34	37.66 ± 2.53	16.18 ± 1.15	36.92 ± 2.51	17.99 ± 1.74
Collagen	1.66 ± 0.55	6.71 ± 1.90 ^#^	2.43 ± 1.26	13.03 ± 5.04 ^#,^*	2.87 ± 1.90	11.06 ± 5.1 ^#,^*

* *p* ≤ 0.05 versus MCD 2 wk; ^$^ *p* ≤ 0.05 versus MCD 4 wk; ^#^ *p* ≤ 0.05 versus respective control. MMPs: DO/mg protein; TIMPs and RECK: A.U./Tubulin; TBARS and GSH: nmoL/mg protein; ROS: A:U; collagen, autofluorescence intensity as A.U.; weeks (wk).

**Table 3:** Correlation between liver Zn and Fe versus MMPs, TIMPs, RECK, TBARS, ROS and GSH.

	Zn		Fe	
	r/rs	*p*	r/rs	*p*
MMP-2	−0.146 rs	ns	0.366 rs	0.023
MMP-9	0.222 rs	ns	0.508 rs	0.002
TIMP-1	0.348 r	0.040	−0.526 r	0.0008
TIMP-2	0.263 rs	ns	−0.559 rs	0.0003
RECK	−0.581 rs	0.001	0.360 rs	0.047
TBARS	−0.485 rs	0.022	0.474 rs	0.022
ROS	−0.478 rs	0.028	0.518 rs	0.013
GSH	0.416 rs	ns	−0.606 rs	0.005

r: Pearson’s correlation coefficient; rs: Spearman’s rank correlation coefficient; ns: *p* value not significant.

**Table 4:** Correlation between liver MMPs versus TBARS, ROS and GSH.

	MMP-2		MMP-9	
	rs	*p*	rs	*p*
TBARS	0.479 rs	0.018	0.578 rs	0.005
ROS	0.613 rs	0.019	0.288 rs	ns
GSH	−0.522 rs	0.018	−0.521 rs	0.026

rs: Spearman’s rank correlation coefficient; ns: *p* value not significant.

**Table 5:** Changes in serum MMPs, IL-1beta, IL-6 and TNF-alpha using rats fed with MCD diet for 2-, 4- and 8-week.

	C 2 wk	MCD 2 wk	C 4 wk	MCD 4 wk	C 8 wk	MCD 8 wk
MMP-2	733.0 ± 22.1	680.4 ± 32.4	758.4 ± 24.1	683.5 ± 66.1	665.1 ± 68.3	694.2 ± 66.0
MMP-9	312.7 ± 10.9	312.0 ± 9.2	327.2 ± 11.2	296.3 ± 22.2	402.7 ± 23.8	295.9 ± 45.3
IL-1beta	23.68 ± 5.30	80.38 ± 30.16 ^#^	25.81 ± 2.73	42.31 ± 2.60 ^#^	29.73 ± 1.35	53.11 ± 7.98 ^#^
IL-6	36.98 ± 4.88	20.28 ± 6.81 ^#^	23.89 ± 6.96	22.95 ± 1.25	40.76 ± 5.55	119.07 ± 46.49 ^#,^*^,$^
TNF-alpha	28.70 ± 2.09	33.79 ± 2.06	26.73 ± 0.52	35.27 ± 3.08	25.97 ± 2.20	35.31 ± 2.57

* *p* ≤ 0.05 versus MCD 2 wk; ^$^ *p* ≤ 0.05 versus MCD 4 wk; ^#^ *p* ≤ 0.05 versus respective control. MMP-2 and MMP-9: DO/mg protein; IL-1beta, IL-6 and TNF-alpha: pg/mL; weeks (wk).

**Table 6:** Correlation between serum Zn and Fe versus MMPs, IL-1beta, IL-6 and TNF-alpha.

	Zn		Fe	
	r/rs	*p*	r/rs	*p*
MMP-2	0.218 r	ns	0.207 r	ns
MMP-9	0.371 r	ns	−0.310 r	ns
IL-1beta	−0.670 rs	0.0009	0.576 rs	0.008
IL-6	0.026 rs	ns	0.332 rs	ns
TNF-alpha	−0.470 r	0.03	0.742 r	0.0002

r: Pearson’s correlation coefficient; rs: Spearman’s rank correlation coefficient; ns: *p* value not significant.

**Table 7:** Changes in fatty acids in rats fed MCD diet.

	C 2 wk	MCD 2 wk	C 4 wk	MCD 4 wk	C 8 wk	MCD 8 wk
C 16:0, palmitic acid	25.5 ± 1.5	23.6 ± 0.8	20.9 ± 0.5	21.9 ± 0.9	21.1 ± 1.6	21.0 ± 0.5 *
C 18:0, stearic acid	15.2 ± 1.8	6.7 ± 0.4 ^#^	16.2 ± 0.8	6.8 ± 0.2 ^#^	17.1 ± 0.8	6.1 ± 0.3 ^#^
C 18:1 n7, vaccenic acid	3.3 ± 0.7	2.2 ± 0.2	3.2 ± 0.3	2.0 ± 0.1 ^#^	4.6 ± 0.3	1.8 ± 0.1 ^#,^*
C 18:1, n9 oleic acid	17.1 ± 1.7	23.2 ± 0.9 ^#^	17.8 ± 1.0	21.6 ± 0.9	17.3 ± 1.2	20.3 ± 0.9
C 18:2 n6, linoleic acid	20.5 ± 1.3	30.3 ± 0.5 ^#,$^	19.0 ± 0.6	28.2 ± 0.4 ^#,^*	16.6. ± 0.7	27.0 ± 0.3 ^#,^*
C 20:4 n6, arachidonic acid	11.5 ± 1.2	6.0 ± 0.6 ^#^	13.0 ± 0.9	7.7 ± 0.4 ^#^	17.8 ± 1.6	8.9 ± 0.6 ^#,^*
C 22:4 n6 docosatetraenoic acid	0.5 ± 0.1	1.1 ± 0.1 ^#^	0.8 ± 0.1	2.3 ± 0.3 ^#^	1.0 ± 0.3	2.8 ± 0.1 ^#,^*
C 22:5 n3, docosapentaenoic acid	0.4 ± 0.1	0.8 ± 0.1 ^#^	0.4 ± 0.1	0.3 ± 0.1 *	0.3 ± 0.1	0.2 ± 0.1 *
C 22:6 n3, docosahexaenoic acid	1.5 ± 0.2	0.8 ± 0.1 ^#^	0.9 ± 0.1	2.2 ± 0.2 *	2.0 ± 0.3	4.0 ± 0.3 ^#,^*^,$^

* *p* ≤ 0.05 versus MCD 2 wk; ^$^ *p* ≤ 0.05 versus MCD 4 wk; ^#^ *p* ≤ 0.05 versus respective control. Fatty acids are expressed as a percentage of total fatty acids in the liver; weeks (wk).

**Table 8:** Correlation between liver Zn and Fe versus fatty acids.

	Zn		Fe	
	r/rs	*p*	r/rs	*p*
C 16:0	0.042 rs	ns	0.079 rs	ns
C 18:0	0.594 rs	0.0004	−0.507 rs	0.003
C 18:1 n7	0.491 rs	0.005	−0.336 rs	0.05
C 18:1 n9	−0.244 r	ns	0.283 r	ns
C 18:2 n6	−0.376 rs	0.04	0.386 rs	0.03
C 20:4 n6	0.460 rs	0.009	−0.299 rs	ns
C 22:4 n6	−0.653 rs	0.0001	0.540 rs	0.002
C 22:5 n3	0.243 rs	ns	−0.037 rs	ns
C 22:6 n3	−0.220 rs	ns	0.067 rs	ns

r: Pearson’s correlation coefficient; rs: Spearman’s rank correlation coefficient; ns: *p* value not significant.

## Data Availability

The data presented in this study are available on request from the corresponding author.

## Supplementary Materials

The following supporting information can be downloaded at: https://www.mdpi.com/article/10.3390/ijms23126817/s1.
