# Supplementary material for: MCD Diet Rat Model Induces Alterations in Zinc and Iron during NAFLD Progression from Steatosis to Steatohepatitis
Source: Int J Mol Sci. 2022 Jun 19;23(12):6817. doi: 10.3390/ijms23126817 (PMC9224179; doi:10.3390/ijms23126817)
Supplement: Supplementary file 1 [file ijms-23-06817-s001.zip › ijms-1772102-supplementary.pdf]

Figure S1: Representative Western blots of TIMPs and RECK

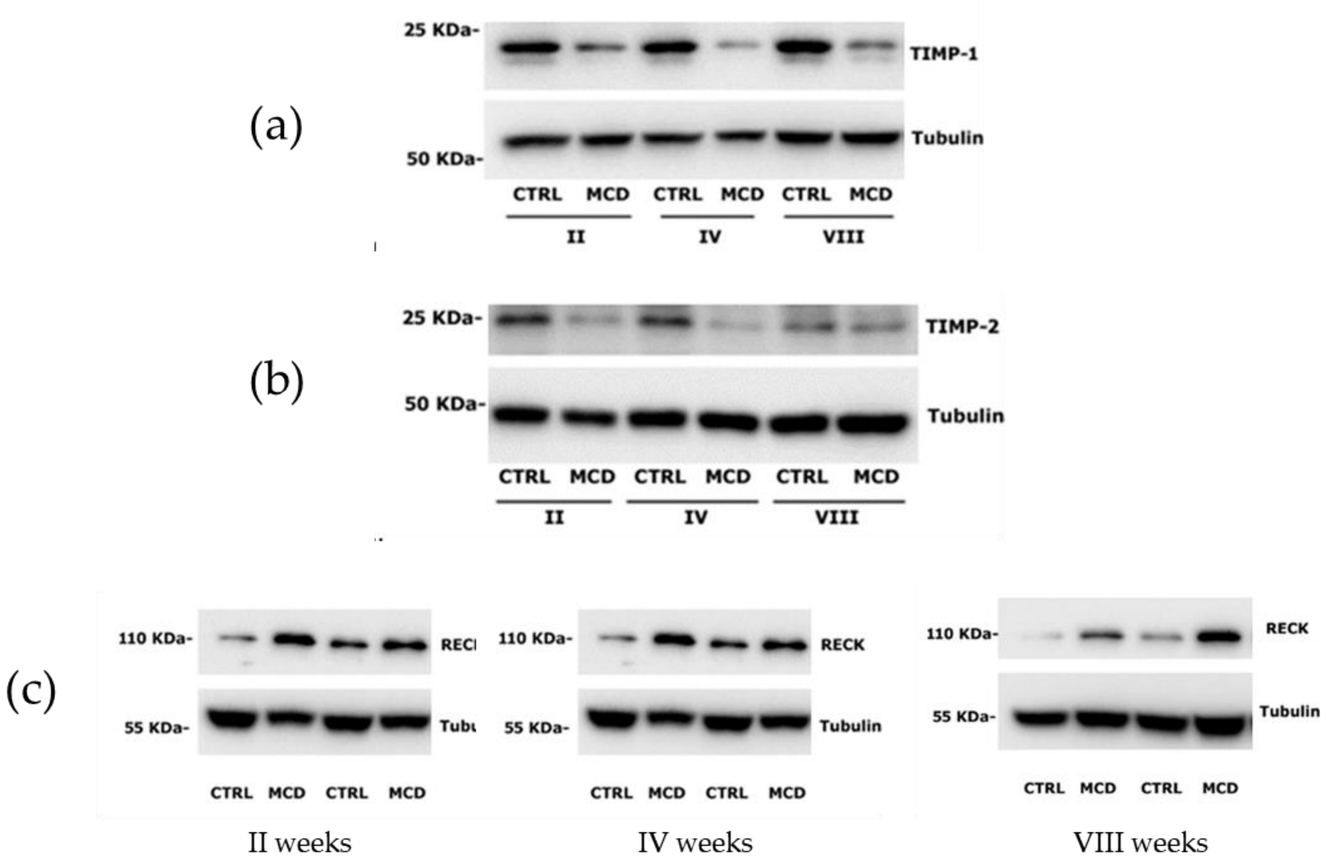

Figure S1. Representative Western blots of TIMP-1 (panel a), TIMP-2 (panel b) and RECK (panel c) determined in livers obtained from rats fed with Control or MCD diet 2, 4 and 8 weeks.
